# Dolodoc, an App to Leverage Self-Management of Chronic Pain: Design, Development, and Implementation Report

**DOI:** 10.2196/71597

**Published:** 2025-08-08

**Authors:** Frederic Ehrler, Julie Guebey, Jessica Rochat, Laetitia Gosetto, Benno Rehberg, Christian Lovis, Aude Molinard-Chenu

**Affiliations:** 1 Division of medical information science, Diagnostic department, University Hospital of Geneva, Gabrielle Perret Gentil 4, Geneva, 1200, Switzerland, 41 765327838; 2 Anesthesiology, University Hospital of Geneva, Geneva, Switzerland; 3 Medicine Faculty, University of Geneva, Geneva, Switzerland

**Keywords:** implementation, chronic pain, mHealth, self-management, health behavior change, user-centered design

## Abstract

**Background:**

Chronic pain affects approximately 19% of the European population and presents major challenges, both in terms of individual impact and the economic burden on health care systems. While clinical expertise remains essential, patient empowerment through self-management tools has become a key component in the long-term management of chronic pain.

**Objective:**

This report describes the development and implementation of Dolodoc, a mobile app designed to support patients with chronic pain in monitoring and managing their condition.

**Methods:**

Developed by a research and development team at the University Hospitals of Geneva, Dolodoc enables users to track their pain across 7 dimensions of daily life. A digital coach provides personalized guidance, drawing from a corpus of over 80 evidence-based recommendations elaborated by clinical experts. The project was conducted over 4 years with the early involvement of stakeholders, including pain specialists and end users, to ensure alignment with user needs. Emphasis was placed on both the scientific validity and accessibility of the recommendations.

**Results:**

The project was completed on time and within budget. The app was made freely available to patients identified as likely to benefit. However, a notable limitation is the absence of predefined key performance indicators to assess the impact of the intervention quantitatively.

**Conclusions:**

This implementation report illustrates how mobile technology can be leveraged in a university hospital context to address the needs of patients with chronic pain and promote self-management. Early and sustained collaboration with stakeholders was instrumental in aligning the solution with both clinical evidence and user expectations.

## Introduction

### Context

Chronic pain is defined by the International Association for the Study of Pain as “an unpleasant sensory and emotional experience associated with, or resembling that associated with, actual or potential tissue damage, that persists over a period of at least three months” [1]. It is a ubiquitous issue affecting about 19% of the European population [2], associated with considerable burden and high health care costs globally [3,4]. Follow-up studies of patients with chronic pain report a persistence rate of approximately 50%‐71% after 1 year [5,6], with high interindividual variability depending on biopsychosocial factors, functional limitations, and comorbidities [7,8]. Over the years, chronic pain treatment has evolved into a multimodal arsenal, including lifestyle changes [9], physical therapy [10], psychotherapy [11], interventional treatments [12], and medications [13]. In addition to pain reduction, integrated follow-up in a multidisciplinary pain center can improve disability and quality of life [14].

Alongside expert health care support, patient-directed strategies have become crucial in addressing long-term pain conditions effectively [15]. With the widespread adoption of mobile devices, smartphone-delivered interventions for pain management have gained prominence in the field of chronic pain care [16].

### Problem Statement

The availability of professional multidisciplinary follow-up is limited and costly. Moreover, the longitudinal evolution of pain and quality of life indicators is not easily gathered by patients and health care professionals. Patient engagement in pain management strategies between appointments is also difficult. [17].

These issues could be addressed by a digital intervention that transmits targeted health information, including behavior change communication such as multimodal chronic pain self-management strategies. It should also allow users to track and share long-term data on pain levels and quality of life. To improve user engagement, this mobile app must be user-centered, intuitive, and inviting.

### Similar Interventions

Dolodoc’s development was inspired by existing mobile apps for chronic pain management [18], which typically focus on one or more of the following objectives: education, monitoring, and management [19]. Unlike these, Dolodoc integrates all 3 objectives into a cohesive, patient-centered system, adding significant value by fostering active patient engagement and collaboration with health care professionals [20]. Key differentiators include an emphasis on quality-of-life reporting and a digital coach that offers personalized activity recommendations and feedback. This helps users identify effective pain management strategies and supports adherence to these strategies through motivational interactions. Furthermore, Dolodoc is specifically tailored for French-speaking users, addressing a linguistic gap in the availability of such tools.

Following the iCHECK-DH (Checklist for the Reporting on Digital Health Implementations; Checklist 1) guidelines for reporting on digital health implementations [21], we describe “Dolodoc,” a mobile app co-designed with patients with chronic pain and health care professionals to monitor pain and quality of life, and provide multimodal self-management strategies that can be experienced and assessed within the mobile app.

## Methods

### Aim and Objectives

Our goal was to develop a system that assists patients with chronic pain in actively managing their condition, providing continuous support, and empowering patients to engage in a personalized approach to improving their well-being.

### Blueprint and Summary

**Figure 1.**
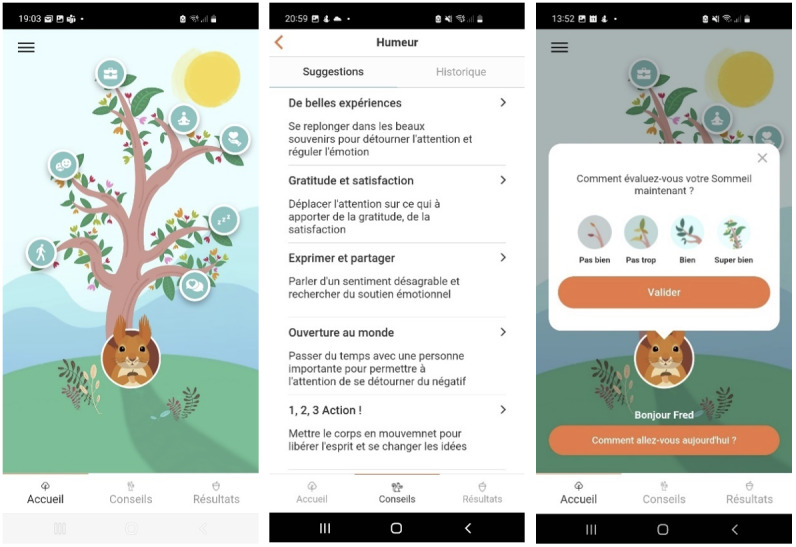
Screenshots of the Dolodoc app.

Dolodoc is a mobile app, in French, that enables users to report their perceptions of pain and the impact of pain on various aspects of their quality of life [22]. The system includes advice as well as activities that patients can plan and assess to identify effective pain management strategies. Dolodoc engages patients through a digital coach that reacts to the planification of activities, based on the strategies included in the app. Additionally, the coach prompts users to regularly report their perceptions of pain and their daily functioning. Furthermore, the app provides an option to share progress with health care professionals, fostering a collaborative approach to managing chronic pain. A gamified metaphorical universe—represented by a tree that flourishes or withers based on the user’s evaluations—aiming to enhance engagement and motivation (Figure 1). Overall, Dolodoc combines elements addressing the 3 objectives that are usually found in digital interventions for chronic pain [19]: education, monitoring, and management.

The development of Dolodoc included 3 primary phases: conception, content creation and validation, and technical implementation. The original concept was fostered in a research and development department combining medical clinical expertise and psychology skills. To ensure user-centered design and relevance, all phases of the app development were elaborated in a user-centered manner, including critical stakeholders such as patients and health care professionals involved in the Geneva Pain Network. A focus group of 10 patients with chronic pain was interviewed about the dimensions of their quality of life that they would relate to chronic pain. Seven dimensions emerged from open discussions in the group: daily activities, mood, work, relaxation, social support, sleep, and intimacy. Based on scientific evidence, a psychologist wrote a first version of multiple advice per dimension. Then, an experienced pain clinician and a member of the hospital communication team revised the advice. The current version of the app contains a database of 84 expert-recommended strategies related to patient-centered dimensions of quality of life. Patients with chronic pain also participated in the conception of the app by providing regular feedback on the prototyped screen as well as on the proposed graphical design of the app. Finally, the app has been reviewed by a user experience designer in order to identify usability problems.

The technical implementation included the development of a mobile app, incorporating gamification and privacy features. The graphic environment of the app was built according to the feedback of the focus group. Indeed, to facilitate engagement, Dolodoc uses a gamified metaphor: a digital tree that flourishes or withers according to the patient’s self-assessment in the 7 dimensions of quality of life.

After a development phase, the app was released on Android and Apple app stores in March 2023. Minor bugs were addressed in several updates until the last release in June 2024.

For deployment, a communication campaign was conducted in January 2024, including printed flyers that were arranged at the pain center as well as billboards that were placed within the hospital and in neighboring high-traffic areas, and targeted social media advertisements, to raise awareness of the app among potential users. These efforts aimed to maximize outreach and ensure adoption among the chronic pain community.

### Technical Design

The Dolodoc app was developed internally to align with the hospital’s strategic goal of creating a cohesive ecosystem of apps tailored to patient needs. The internal development approach ensured seamless integration of hospital-curated advice and positioned the app for future connectivity with the clinical information system, enhancing follow-up for chronic disease patients.

From a technological perspective, coach interactions are currently driven by a predefined rule-based system, meaning that all the coach’s interactions are statically coded. While its current state relies on predefined interactions, there is potential to incorporate large language models to improve personalization and adaptability in the future. The app is free to download and fully owned by the University Hospitals of Geneva (HUG), aligning with their patient-first, noncommercial ethos. This ownership guarantees control over intellectual property and the ability to integrate evolving technologies and data security measures. The app’s open architecture supports long-term alignment with the hospital’s digital health investment roadmap, emphasizing interconnected and user-centric solutions.

### Target

Dolodoc was developed to primarily target French-speaking patients enduring chronic pain. However, since the app is free and available for download by a large population, we foresee that the content of the app (eg, the advice) could also be used by other members of the health system around a chronic pain situation, such as health care professionals and caregivers.

### Data

The app ensures data privacy and security in line with health care data management standards. Dolodoc uses a device-based data governance approach, where all personal data collected by the app is stored locally on the user’s device. Data goes through a lifecycle that includes collection, processing, and storage on the device itself, without being transferred to external servers or cloud-based storage. As such, data ownership remains entirely with the patient, who has full access and control over their information. Data regarding the use of the app is collected anonymously through the Piwik web analytics tool installed on-premises on our infrastructure. Consent for data collection is obtained when the user accepts the disclaimer presented at the initial setup of the app, clearly outlining data handling practices and user responsibilities.

Patients have the option to share their personal data with clinicians in a secure, confidential manner by generating a PDF report directly from the app. Sharing the report is initiated solely at the user’s discretion and responsibility. Given that the data remains on the user’s device, data protection measures are focused on device security, recommending that users follow best practices in device security (eg, password protection and regular software updates).

### Interoperability

The current version of the app is not connected to another system.

### Participating Entities

The Dolodoc app was developed at the HUG, one of Switzerland’s largest university hospitals. This project was carried out within the SIMED (Medical Information Science Department), a research and development service dedicated to research projects and specializing in human-machine interaction. The SIMED leveraged the multidisciplinary competencies of the team, such as ergonomics, psychology, clinical expertise, and technical ones to cover all aspects of the project. Cooperation with pain experts from the Geneva pain network, as well as patients, ensured adequacy of the tool with targeted user needs, as well as compliance with the latest medical evidence. Funding was provided by the Fondation Privée des HUG, a foundation focused on supporting projects enhancing the quality of care at the hospital through private donations. The funds covered expenses related to the app’s conception, development, and design. The project involved pain management specialists and patient-partners to ensure the app’s relevance and efficacy. This funding was sufficient to fully support the implementation phase. The final product and intellectual property will remain under the ownership of HUG after implementation.

### Budget

The development of Dolodoc required an estimated budget of approximately CHF 250,000 (US $250,000). A substantial portion of the budget was allocated to content creation and validation. The budget covers the complete lifecycle of Dolodoc creation and deployment, ensuring a robust foundation for ongoing use and impact.

### Sustainability

Dolodoc operates as an institutional project focused on enhancing patient support for chronic pain management, rather than generating financial profit. The primary goal of the app is to provide long-term value to patients by offering a self-management tool that empowers users and improves quality of life. Sustainability is ensured through institutional backing, where ongoing costs are centered on maintaining the app within the technical framework. This includes updates to ensure compatibility with evolving device operating systems, security enhancements, and periodic content revisions to maintain relevance and accuracy.

### Ethical Considerations

This study did not require approval from an ethics review board, as it did not involve the collection or analysis of identifiable personal health data, nor any clinical experimentation. The development and implementation of Dolodoc were conducted as part of a quality improvement and service design initiative at the HUG, in accordance with Swiss federal guidelines under the Human Research Act (2014), Article 2(1), which exempts projects not involving health-related personal data from ethics approval requirements. During the co-design process, patients participated voluntarily in interviews and usability testing sessions focused on app functionality and content design. No health or sensitive data were recorded, and all feedback was anonymized at the point of collection. Users of the final version of the app consent to anonymous usage data collection through an internal instance of the Piwik analytics platform, as clearly outlined in the initial disclaimer presented upon first use of the app. No participants received any form of compensation. As such, the project complies with institutional and national standards for service-level digital health development outside the scope of research involving human participants.

## Results

### Coverage

The coverage of the implementation is international since it is available on several app stores. Indeed, use analysis showed that Dolodoc was used in 23 different countries. However, Dolodoc is designed in French, and French-speaking countries (Switzerland and France) account for >90% of the users. In Switzerland, for example, 22.8% of the population speaks French as a main language [23].

### Outcome

Since it is publicly available until November 2024, Dolodoc was downloaded 2284 times in total, including Android and Apple stores. The promotional strategy was efficient since it boosted downloads, reaching a maximum of 206/week during the first month of the campaign.

At the pain center, Dolodoc has been included in the multimodal therapeutic strategy that is discussed between patients and pain specialists. It is mentioned in our medical reports, and it is also used in psychotherapy sessions.

Clinical indicators about self-efficacy and quality of life were not systematically collected in our implementation process since the budget did not include a clinical impact study. Hence, a dedicated study should be planned to address the clinical added value of this implementation.

Qualitative feedback was obtained from open discussions with Dolodoc users in the context of a digital navigation [24] follow-up at the pain center. The role of a digital navigator is to help users navigate digital technologies efficiently by guiding, answering questions, exposing all the digital possibilities according to each patient, and fixing technical problems.

Participants reported that they appreciated the variety of recommendations and the way they were presented, which sometimes alleviated helplessness. While the number of strategies was at times overwhelming, users appreciated having alternatives. Considering whether the strategy is “achievable” helped to relieve the guilt of not doing everything without losing motivation. In fact, patients spontaneously reported that the strategies are delivered in a nonjudgmental and informative way, and the variety of strategies was also greatly appreciated, as emphasized in the following quote: “It makes you realize that there are many ways to take care of yourself”. Self-management strategies were considered the app’s main value, even as reminders. The strategies, which were given as a resource, are also applicable outside of painful crises and have general health benefits, which were appreciated. Some weaknesses in the navigability within the app were also reported by participants: the fact that users must actively browse and choose a suitable strategy within the app was perceived as an obstacle to action. The lack of a reminder feature was also noticed as a barrier to use. Indeed, users commented on the importance of motivational aspects in chronic pain self-management that were not entirely fulfilled by Dolodoc.

### Lessons Learned

The involvement of patients and specialists, such as psychologists and pain experts, from the initial stages of conception proved to be a critical success factor in ensuring the app aligned with the needs of its target users. Engaging these stakeholders helped to identify meaningful pain management strategies and ensured that the app addressed real-world challenges faced by individuals with chronic pain. The adoption of a user-centered user interface/user experience design approach further strengthened the solution, incorporating steps like user requirement identification, prototyping, and iterative testing. This ensured that the app was intuitive and functional before technical implementation began. Additionally, trust in the app was bolstered by its development under the sponsorship of a nonprofit health care institution, reassuring users of its credibility and noncommercial intent.

However, generating personalized and relevant advice for patients posed a significant challenge. This required extensive evidence assessment, interviews with end users, and iterative reviews by experts and communication specialists to ensure the advice was both scientifically grounded and comprehensible to users. Budget constraints added another layer of difficulty, as the project was bound by a predefined budget set before finalizing the full specifications. Some features, initially thought to be straightforward, proved much more complex and resource-intensive to implement, underscoring the importance of thorough feasibility assessments during the planning phase. Moreover, the lack of budget for a clinical impact study prevents us from anchoring our implementation on specific evidence about Dolodoc, although global evidence supports the effectiveness of self-management apps for chronic pain [16].

### Unintended Consequences

Although pain specialists at the pain center were informed about and convinced of the utility of adding Dolodoc to their therapeutic arsenal, it took time for them to integrate this novel tool into their regular clinical practice. The engagement of patients in using the app also turned out to be a limitation. Indeed, we noticed that even when the pain specialist provided information about Dolodoc during a medical visit, most patients did not use the app until the following visit. These 2 pitfalls are part of a well-described phenomenon regarding the implementation of digital health interventions [25]. It could perhaps be addressed by integrating digital navigators into our clinical practice.

## Discussion

Dolodoc’s implementation demonstrates the transformative potential of mobile health apps in addressing chronic pain management challenges. By equipping patients with self-monitoring tools and personalized strategies, Dolodoc addresses key barriers like limited access to multidisciplinary care and low patient engagement between visits.

User-centered design and stakeholder engagement were critical to the app’s success. Ongoing collaboration with patients with chronic pain, clinicians, and psychologists ensured usability and relevance. Gamification and a strong privacy framework also boosted trust and engagement.

Users reported benefits such as reduced feelings of inadequacy and better access to new strategies. However, they also emphasized the difficulty of finding suitable strategies during episodes of intense pain, highlighting the need to improve accessibility and navigation during pain crises, consistent with previous findings [26]. The lack of predefined key performance indicators and budgetary provisions for a clinical impact study highlights a significant limitation, underscoring the need for future implementations to include robust evaluation frameworks. These would validate the clinical efficacy of digital tools and provide insights for iterative improvements.

Looking ahead, Dolodoc presents opportunities to advance chronic pain management on multiple fronts. Integration with hospital information systems could enhance the app’s utility in clinical settings, supporting coordinated care. Additionally, leveraging technologies such as large language models could enable more dynamic and personalized patient interactions. However, overcoming challenges in adoption—both among clinicians and patients—remains essential. Strategies like integrating digital health navigators and expanding awareness campaigns could boost utilization rates and long-term engagement.

Ultimately, Dolodoc exemplifies how mobile health tools can improve self-management and quality of life for patients with chronic pain. Broader use and integration of evaluation frameworks could lead to significant improvements in pain care and serve as a model for future digital health innovations.

## Supplementary material

10.2196/71597 Checklist 1 iCHECK-DH (Checklist for the Reporting on Digital Health Implementations) checklist.

## Abbreviations

HUG: University Hospitals of Geneva
iCHECK-DH: Checklist for the Reporting on Digital Health Implementations
SIMED: Medical Information Science Department
