# Supplementary material for: Dolodoc, an App to Leverage Self-Management of Chronic Pain: Design, Development, and Implementation Report
Source: JMIR Med Inform. 2025 Aug 8;13:e71597. doi: 10.2196/71597 (PMC12334107; doi:10.2196/71597)
Supplement: Checklist 1 [file medinform-v13-e71597-s001.pdf]

Multimedia Appendix 1. Checklist of iCHECK-DH guidelines. iCHECK-DH: Guidelines and Checklist for the Reporting on Digital Health Implementations.

SECTION		ITEM	DESCRIPTION
**TITLE**	1	Title (M[1])	Dolodoc, an App to leverage self-management of Chronic Pain: Design, Development, and Implementation Report
**ABSTRACT**	2	Abstract (M)	Chronic pain affects approximately 19% of the European population and poses significant challenges, both in terms of individual suffering and the financial burden on healthcare systems worldwide. While healthcare provider expertise is critical, empowering patients with self-management tools has become increasingly important for addressing long-term pain effectively. This report details the development and implementation of Dolodoc, a mobile application designed by an R&D team at the University Hospitals of Geneva. The app allows users to monitor their pain and its impact across seven dimensions of daily life. A virtual coach guides users through the process, offering tailored advice from a corpus of over 80 evidence-based recommendations created by experts.<br>The project, which lasted four years, was completed within budget and highlights the importance of early stakeholder involvement, including pain experts and end-users, to ensure the solution met user needs. Significant efforts were dedicated to ensuring the recommendations were both evidence-based and accessible. While the app has been freely offered to patients who may benefit, one limitation of this initiative is the lack of predefined Key Performance Indicators (KPIs) to measure its success quantitatively.<br>This implementation report serves as a real-world example of leveraging mobile technology in a university hospital setting to address the needs of chronic pain patients and empower them in managing their condition
**INTRODUCTION**	3	Context (M)	Chronic pain is defined by the International Association for the Study of Pain (IASP) as "an unpleasant sensory and emotional experience associated with, or resembling that associated with, actual or potential tissue damage, that persists over a period of at least three months". It is a ubiquitous issue affecting about 19% of the European population that is associated with considerable burden and massive costs to healthcare systems worldwide. Follow-up studies of chronic pain patients report a persistence rate of approximately 50-71 % after one year, encompassing high interindividual variability depending on biopsychosocial factors, functional limitations, and comorbidities. Over the years, the treatment of chronic pain has evolved into a multimodal arsenal, including among others lifestyle changes, physical therapy, psychotherapy, interventional treatments and medications. In addition to reducing pain, an integrated follow-up in a multidisciplinary pain center can yield improvements in disability and quality of life.<br>Alongside expert support from healthcare providers, patient-directed strategies have become crucial in addressing long-term pain conditions effectively. With the widespread adoption of mobile devices in recent years, pain management interventions delivered via smartphone applications have gained prominence in the field of chronic pain care.
	4	Problem statement (M)	Chronic pain patients and professionals face many health system challenges: overall the availability of an appropriate professional multidisciplinary follow-up for chroni pain is limited and costly. Moreover, the longitudinal evolution of pain and quality o life indicators are not easily gathered by patients and healthcare professionals. Finally it is difficult to engage patients into pain management strategies between healthcar appointments.<br>These issues could be addressed by the development of a digital intervention that firstl transmits targeted health information, including behaviour change communicatio such as multimodal chronic pain self-management strategies. Secondly this digital too should enable users to track and share long-term data regarding their pain levels an overall quality of life. To improve user engagement, this mobile application should be developed in a user-centred manner, featuring an inviting and intuitive interface.
	5	Similar Interventions	The development of Dolodoc was inspired by existing mobile applications designed for chronic pain management, which typically focus on one or more of the following

[1] M: Mandatory item

		(M)	objectives: education, monitoring, and management. Unlike these applications, Dolodoc integrates all three objectives into a cohesive, patient-centered system, adding significant value by fostering active patient engagement and collaboration with healthcare professionals. Key differentiators include its emphasis on quality-of-life reporting and its interactive virtual coach, which provides personalized activity recommendations and feedback. Potentially, this feature not only empowers users to identify effective pain management strategies but also supports adherence to these strategies through motivational interactions. Furthermore, Dolodoc is specifically tailored for French-speaking users, addressing a linguistic gap in the availability of such tools. By prompting regular reporting on pain and daily functioning and enabling data sharing with healthcare providers, Dolodoc establishes a comprehensive and collaborative approach to chronic pain management that differentiates it from existing solutions.<br>Following the iCHECK-DH guidelines for the reporting on digital health implementations, we will describe "Dolodoc", a mobile application (app) that was designed with chronic pain patients and healthcare professionals, aiming at monitoring pain and quality of life, and providing multimodal self-management strategies that can be experienced and assessed within the mobile application.
**METHODS**	6	Aims and Objectives (M)	Our goal was to develop a system that assists patients with chronic pain in actively managing their condition, providing continuous support, and empowering patients to engage in a personalized approach to improving their well-being.
	7	Blueprint summary (M)	Dolodoc is a patient-centered mobile app, in French, that enables users to report their perceptions of pain and the impact of pain on various aspects of their quality of life. The system includes advice as well as activities that patients can plan and assess, to identify effective pain management strategies. Dolodoc engages patients through a virtual coach that reacts to the planification of activities, based on the strategies included in the app. Additionally, the coach prompts users to regularly report their perceptions of pain and their daily functioning. Furthermore, the app provides an option to share progress with healthcare professionals, fostering a collaborative approach to managing chronic pain. A gamified metaphorical universe—represented by a tree that flourishes or withers based on the user's evaluations—enhances engagement and motivation. Overall, Dolodoc combines elements addressing the 3 objectives that are usually found in digital interventions for chronic pain : education, monitoring and management.<br>The development of Dolodoc included three primary phases: conception, content creation and validation, and technical implementation. The original concept was fostered in an R&D department combining medical clinical expertise and psychology skills. To ensure user-centered design and relevance, all phases of the app development were elaborated in a user-centered manner, including critical stakeholders such as patients and healthcare professionals involved in the Geneva Pain Network. A focus-group of 10 chronic pain patients were interviewed about the dimensions in their quality of life that they would relate to chronic pain. Seven dimensions emerged from open discussions in the group: daily activities, mood, work, relaxation, social support, sleep and intimacy. Based on scientific evidence, a psychologist wrote a first version of multiple advice per dimension. Then, an experienced pain clinician and a member of the hospital communication team revised the advice. The current version of the app contains a database of 84 expert-recommended strategies related to patient-centered dimensions of quality of life.<br>Technical implementation included the development of the mobile app, incorporating gamification and privacy features. The graphic environment of the app was built according to the feedback of the focus group. Indeed, to facilitate engagement, Dolodoc employs a gamified metaphor: a virtual tree that flourishes or withers according to the patient's self-assessment in the seven dimensions pf quality of life.<br>After a development phase, the app was released on Android and Apple app stores in March 2023. Minor bugs were addressed in several updates until the last release in June 2024<br>For deployment, a communication campaign was conducted in January 2024, including printed flyers that were arranged at the pain center as well as billboards that were placed within the hospital and in neighboring high-traffic areas, and targeted social media advertisements, to raise awareness of the app among potential users. These efforts aimed to maximize outreach and ensure adoption among the chronic pain community.

8	Technical Design (M)	The Dolodoc application was developed internally to align with the hospital's strategic goal of creating a cohesive ecosystem of apps tailored to patient needs. The internal development approach ensured seamless integration of hospital-curated advice and positioned the app for future connectivity with the clinical information system, enhancing follow-up for chronic disease patients.<br><br>From a technological perspective, coach interactions are currently driven based on a predefined rule-based system, meaning that all the coach's interactions are statically coded. While its current state relies on predefined interactions, there is potential to incorporate large language models (LLMs) to improve personalization and adaptability in the future. The app is free to download and fully owned by the University Hospitals of Geneva (HUG), aligning with their patient-first, non-commercial ethos. This ownership guarantees control over intellectual property and the ability to integrate evolving technologies and data security measures. The app's open architecture supports long-term alignment with the hospital's digital health investment roadmap, emphasizing interconnected and user-centric solutions.
9	Target (M)	Dolodoc was developed to primarily target French-speaking patients enduring chronic pain. However, since the app is free and available for download by a large population, we foresee that the content of the app (e.g. the advice) could be also used by other members of the health system around a chronic pain situation such as healthcare professionals and caregivers.
10	Data (M)	The app ensures data privacy and security in line with healthcare data management standards. Dolodoc employs a device-based data governance approach, where all personal data collected by the app is stored locally on the user's device. Data goes through a lifecycle that includes collection, processing, and storage on the device itself, without being transferred to external servers or cloud-based storage. As such, data ownership remains entirely with the patient, who has full access and control over their information. Data regarding the usage of the app are collected anonymously through the Piwik™ web analytics tool installed on-premises on our infrastructure. Consent for data collection is obtained when the user accepts the disclaimer presented at the initial setup of the app, clearly outlining data handling practices and user responsibilities.<br>Patients have the option to share their personal data with clinicians in a secure, confidential manner by generating a PDF report directly from the app. Sharing the report is initiated solely at the user's discretion and responsibility. Given that the data remains on the user's device, data protection measures are focused on device security, recommending that users follow best practices in device security (e.g., password protection, regular software updates)
11	Interoperability (M)	The current version of the app is not connected to another system.
12	Participating entities (M)	The Dolodoc application was developed at the University Hospitals of Geneva (HUG), one of Switzerland's largest university hospitals. This project was carried out within the SIMED (Medical Information science department) department, an R&D service dedicated to research projects and specializing in human-machine interaction. The SIMED leveraged the multidisciplinary competences of the team, such as ergonomics, psychology, clinical expertise and technical one to covers all aspects of the project. Cooperation with pain experts from the Geneva pain network as well as patients ensured adequation of the tool with targeted user need as well as compliance to the last medical evidence. Funding was provided by the Fondation Privée des HUG, a foundation focused on supporting projects enhancing the quality of care at the hospital through private donations. The funds covered expenses related to the app's conception, development, and design. The project involved pain management specialists and patient-partners to ensure the app's relevance and efficacy. This funding was sufficient to fully support the implementation phase. The final product and intellectual property will remain under the ownership of HUG after implementation.

	13	Budget Planning (M)	The development of Dolodoc required an estimated budget of approximately 250,000 CHF. A substantial portion of the budget was allocated to content creation and validation. The budget covers the complete lifecycle of Dolodoc creation and deployment, ensuring a robust foundation for ongoing use and impact.
	14	Sustainability (M)	Dolodoc operates as an institutional project focused on enhancing patient support for chronic pain management, rather than generating financial profit. The primary goal of the app is to provide long-term value to patients by offering a self-management tool that empowers users and improves quality of life. Sustainability is ensured through institutional backing, where ongoing costs are centered on maintaining the app within the technical framework. This includes updates to ensure compatibility with evolving device operating systems, security enhancements, and periodic content revisions to maintain relevance and accuracy.
**RESULTS**	15	Coverage (M)	The coverage of the implementation is international since it is available on several app stores. Indeed, usage analysis showed that Dolodoc was used in 23 different countries. However, Dolodoc is designed in French and French-speaking countries (Switzerland and France) account for > 90% of the users. In Switzerland for example, 22.8% of the population speak French as a main language23.
	16	Outcomes (M)	Since it is publicly available until Novembre 2024, Dolodoc was downloaded 2284 times in total, including Android and Apple stores. The promotional strategy was efficient since it boosted downloads, reaching a maximum of 206/week during the first month of the campaign.<br>At the pain center, Dolodoc has been included in the multimodal therapeutic strategy that is discussed between patients and pain specialists. It is mentioned in our medical reports, and it is also used in psychotherapy sessions.<br>Following our sustainability plan, a first update has been released in june 2024, correcting minor bugs.<br>The primary goal of the app is to provide long-term value to patients by offering a self-management tool that empowers users and improves quality of life. This tool is now available for the chronic pain French-speaking community through the design, development, and implementation of Dolodoc. Clinical indicators about self-efficacy and quality of life were, however, not systematically collected in our implementation process since the budget did not include a clinical impact study. Hence, a dedicated study should be planned to address the clinical added value of this implementation.
	17	Lessons learned (M)	The involvement of patients and specialists, such as psychologists and pain experts, from the initial stages of conception proved to be a critical success factor in ensuring the app aligned with the needs of its target users. Engaging these stakeholders helped to identify meaningful pain management strategies and ensured that the app addressed real-world challenges faced by individuals with chronic pain. The adoption of a user-centered UI/UX design approach further strengthened the solution, incorporating steps like user requirement identification, prototyping, and iterative testing. This ensured that the app was intuitive and functional before technical implementation began. Additionally, trust in the app was bolstered by its development under the sponsorship of a non-profit healthcare institution, reassuring users of its credibility and non-commercial intent.<br>However, generating personalized and relevant advice for patients posed a significant challenge. This required extensive evidence assessment, interviews with end-users, and iterative reviews by experts and communication specialists to ensure the advice was both scientifically grounded and comprehensible to users. Budget constraints added another layer of difficulty, as the project was bound by a predefined budget set before finalizing the full specifications. Some features, initially thought to be straightforward, proved much more complex and resource-intensive to implement, underscoring the importance of thorough feasibility assessments during the planning phase. Moreover, the lack of budget for a clinical impact study prevents us to anchor our implementation on specific evidence about Dolodoc, although global evidence is supporting the effectiveness of self-management applications for chronic pain16.
	18	Unintended consequences (NM[2])	Although pain specialists at the pain center were informed about and convinced of the utility of adding Dolodoc to their therapeutic arsenal, it took time for them to integrate this novel tool into their regular clinical practice. The engagement of patients in using the app also turned out to be a limitation. Indeed, we noticed that

[2] NM : Non-mandatory item

			even when the pain specialist provided information about Dolodoc during a medical visit, most patients did not use the app until the following visit. These two pitfalls are part of a well-described phenomenon regarding the implementation of digital health interventions. It could perhaps be addressed by integrating digital navigators into our clinical practice.
**DISCUSSION**	19	Conclusion (M)	The implementation of Dolodoc underscores the transformative potential of mobile health applications in addressing chronic pain management challenges. By empowering patients with tools for self-monitoring and providing personalized, evidence-based strategies, Dolodoc addresses key barriers in traditional pain management approaches, such as limited access to multidisciplinary care and challenges in maintaining patient engagement between medical appointments. These innovations highlight the potential of digital interventions to bridge gaps in chronic pain care, especially within French-speaking populations where such resources are scarce.<br>The successful development of Dolodoc demonstrates the critical role of user-centered design and stakeholder involvement. Early and consistent collaboration with patients, clinicians, and psychologists ensured the app's relevance, usability, and effectiveness in addressing patient needs. Additionally, the gamification element and privacy-first approach further enhance user trust and engagement. However, the lack of predefined key performance indicators (KPIs) and budgetary provisions for a clinical impact study highlights a significant limitation, underscoring the need for future implementations to include robust evaluation frameworks. These would validate the clinical efficacy of digital tools and provide insights for iterative improvements.<br>Looking ahead, Dolodoc presents opportunities to advance chronic pain management on multiple fronts. Integration with hospital information systems could enhance the app’s utility in clinical settings, supporting coordinated care. Additionally, leveraging technologies such as large language models could enable more dynamic and personalized patient interactions. However, overcoming challenges in adoption—both among clinicians and patients—remains essential. Strategies like integrating digital health navigators and expanding awareness campaigns could boost utilization rates and long-term engagement.<br>Ultimately, Dolodoc exemplifies the potential for mobile health solutions to enhance patient empowerment and quality of life in chronic pain management. Expanding such tools globally and integrating comprehensive evaluation frameworks could catalyze widespread improvements in pain care and set benchmarks for future digital health innovations..
**GENERAL**	20	General (NM)	N/A
